# Sensitivity Analysis of Influencing Factors and Two-Stage Prediction of Frost Resistance of Active-Admixture Recycled Concrete Based on Grey Theory–BPNN

**DOI:** 10.3390/ma17081805

**Published:** 2024-04-14

**Authors:** Chun Fu, Ming Li

**Affiliations:** 1 School of Civil Engineering, Liaoning Petrochemical University, Fushun 113001, China; 2 School of Civil Engineering, Shenyang Jianzhu University, Shenyang 110168, China; cemli@sjzu.edu.cn

**Keywords:** sensitivity analysis, recycled concrete, grey theory, BPNN

## Abstract

Sensitivity analysis of influencing factors on frost resistance is carried out in this paper, and a two-stage neural network model based on grey theory and Back Propagation Neural Networks (BPNNs) is established for the sake of predicting the frost resistance of active-admixture recycled concrete quickly and accurately. Firstly, the influence degree of cement, water, sand, natural aggregate, recycled aggregate, mineral powder, fly ash, fiber and air-entraining agent on the frost resistance of active-admixture recycled-aggregate concrete was analyzed based on the grey system theory, and the primary and secondary relationships of various factors were effectively distinguished. Then, the input layer of the model was determined as cement, water, sand, recycled aggregate and air-entraining agent, and the output layer was the relative dynamic elastic modulus. A total of 120 datasets were collected from the experimental data of another author, and the relative dynamic elastic modulus was predicted using the two-stage BPNN prediction model proposed in this paper and compared with the BPNN prediction results. The results show that the proposed two-stage BPNN model, after removing less-sensitive parameters from the input layer, has better prediction accuracy and shorter run time than the BPNN model.

## 1. Introduction

Nowadays, the global urbanization and industrialization process has increased the demand for natural concrete, which has led to the destruction of the natural environment, energy consumption and environmental pollution and other problems that cannot be ignored [1,2,3]. Making matters worse, the waste concrete generated by the renovation and reconstruction of urban buildings is even worse for environmental protection, and even causes a huge waste of resources. Recycled-aggregate concrete came into being at the right moment. Recycled aggregate is obtained by crushing and screening waste concrete, and it is made of recycled-aggregate concrete instead of natural aggregate, which can effectively realize the reuse of waste concrete, which can effectively alleviate the dilemma of short supply of sand and stone, and also meet the requirements of today’s society for green concrete, and play a positive role in promoting the sustainable development of building resources and the environment [4,5]. It is without doubt that the recycling of waste concrete is a win–win solution, and has extensive application prospects [6,7,8].

Generally, the amount of bonding mortar and the quality of the original concrete have a significant effect on the performance of recycled concrete; the increase in recycled-aggregate content and water–cement ratio will reduce the durability of concrete [9]. Adding various industrial wastes such as fly ash and mineral powder into concrete as active admixtures can not only reduce the amount of cement, but also improve the frost resistance of concrete, which has become an important direction of the development of building science [10,11,12,13]. Cui et al. [14] studied the effect of waste polypropylene fiber on the frost resistance of recycled-aggregate concrete, and the results showed that waste fiber could improve the frost resistance of recycled concrete. Abed et al. [15] studied the chloride ion migration, concrete impermeability and freeze–thaw resistance of self-compacting high-performance concrete mixed with unprocessed waste fly ash, waste perlite powder and waste honeycomb concrete, respectively. The results showed that pozolanic activity and amorphous waste-powder materials can improve the durability of concrete under aggressive environments. Li et al. [16] conducted an experimental study on the frost resistance of recycled-aggregate concrete made of iron tailings, and the results showed that the combination of 30% iron tailings and 30% recycled-aggregate concrete had good frost resistance. The micro-analysis showed that when an appropriate amount of iron tailings was used, the pore structure of and frost resistance of recycled concrete were improved. Jain et al. [17] studied the effects of different alternative levels of waste glass powder and granite powder on the durability of concrete, and the results showed that the durability of concrete was significantly improved when 15% of waste glass powder and 30% of granite powder were contained.

The reason for the poor frost resistance of recycled-aggregate concrete is clear. The micro-cracks caused by the damage accumulation and crushing process of the original concrete not only increase the water absorption rate of the recycled concrete, but also provide a new channel for water to penetrate the concrete, which makes the recycled concrete more prone to freeze–thaw damage. So, it is necessary to evaluate the frost resistance of recycled-aggregate concrete before promoting it in cold areas [18,19]. At present, the commonly used evaluation indexes for frost resistance of concrete mainly include mass loss rate, compressive strength loss rate and relative dynamic elastic modulus, etc. Due to the fact that measurement results of relative dynamic elastic modulus are accurate and do not need to destroy the specimen, and can sensitively reflect the damage inside the concrete, it is widely used by scholars [20,21]. It is concluded from the above analysis that most of the current studies are mainly based on test and theoretical analysis, and a set of practical, reasonable and complete evaluation methods have not been established for the frost resistance of recycled concrete based on relative dynamic elastic modulus. How to carry out effective data mining on the collected information, so as to carry out scientific risk identification, early warning, prevention and control of concrete durability has become an urgent problem to be solved.

In recent years, machine learning methods have attracted more and more attention [22,23]. Machine learning has the advantages of self-organization, self-learning, and the ability to quickly and accurately reflect the relationships between a variety of influential factors. Many scholars have tried to use neural network models to predict some properties of concrete, and have obtained satisfactory results. Hosseinzadeh et al. [24] focused their research on the prediction of mechanical properties of fly ash recycled-aggregate concrete based on a machine learning algorithm, and the results showed that the accuracy of XGBoost algorithm in the prediction of compressive and tensile strength was higher than that of random forest algorithm, about 0.95. Concha [25] used a neural network to predict the carbonization depth of recycled-aggregate concrete, and the prediction results showed that the prediction model could provide better prediction results even if there was ambiguity in the data, and the results could be used to evaluate the health status of recycled-aggregate concrete structures. Huang et al. [26] used a convolutional neural network (CNN) to predict the compressive strength of mixed-fiber-reinforced recycled-aggregate concrete. The results showed that the CNN prediction model had good prediction accuracy, and the average relative error and maximum relative error of the prediction results were 1.98% and 4.12%, respectively. Boudali et al. [27] used an artificial neural network (ANN) to predict the compressive strength of recycled self-compressive concrete by taking binder content, water–binder ratio, recycled concrete aggregate content, fly ash content, recycled concrete powder content and curing time as input vectors. Dong et al. [28] used the Grey Wolf optimizer (GWO) to enhance the BPNN and established an optimization model for finding the best mix-ratio of ecological concrete. B K A et al. [29] used an ANN to predict the compressive strength of recycled concrete and obtained satisfactory accuracy.

In general, increasing the number of input variables of a neural network can result in better prediction results, and the condition of input variables has a good correlation with the output variables. However, some inputs may be irrelevant or contribute no information to the output, and may introduce system noise, fool the training algorithm and degrade the performance of the model [30,31]. Therefore, there is an urgent problem to be solved about the sensitivity analysis of input variables. Based on the above analysis, this paper attempted to apply grey correlation degree and a BPNN to predict the frost resistance of recycled-aggregate concrete with active admixture, in order to provide a new idea for the frost resistance prediction of recycled concrete.

## 2. Mix Proportion of Active-Admixture Recycled Concrete

According to [32], with the increase in freeze–thaw times, the relative dynamic elastic modulus of recycled aggregate concrete decreases gradually; it means that the inside freeze–thaw damage of concrete increases gradually and, thus, the conclusion is inevitable that the number of freeze–thaw cycles has the greatest and most continuous influence on the frost resistance of concrete. Therefore, the frost resistance of active-admixture recycled concrete is related to 10 factors, including the quality of cement (Ordinary Portland cement 42.5 grade), water, sand, natural coarse aggregate, recycled aggregate, mineral powder, fly ash, air-entraining agent and fiber in 1 m^3^ concrete, and the number of freeze–thaw cycles. It should be noted that in view of the importance of the number of freeze–thaw cycles on the influence of frost resistance, only the sensitivity analysis of the remaining nine concrete mix components is required. For the input and output vectors of an artificial neural network (ANN), all data must be normalized. Table 1 shows the range of variation for each variable.

## 3. Two-Stage Prediction Model

### 3.1. Model Description

**Figure 1:**
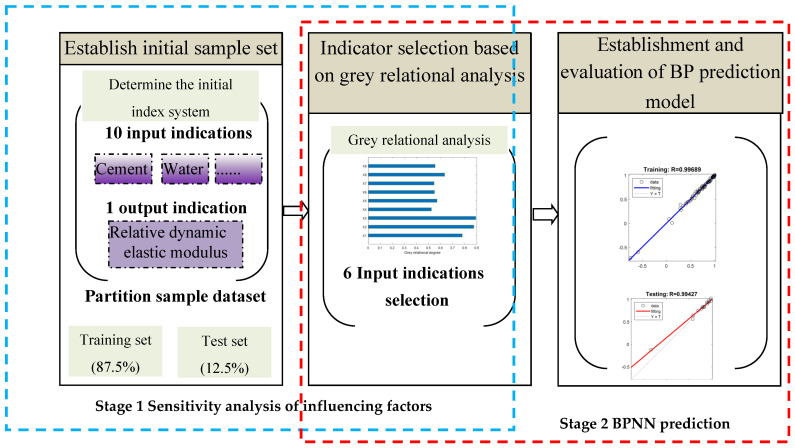
Two−stage frost resistance prediction model based on grey theory and BPNN.

A two-stage prediction model is proposed in this paper, aiming at predicting the frost resistance of active-admixture recycled concrete based on grey influencing factors sensitivity analysis and a BPNN. The method can be divided into two stages and three steps: initial indicator determination, indicator screening, BPNN prediction model establishment and evaluation, as shown in Figure 1.

### 3.2. Establishment of the Initial Sample Set

The database used in this paper is 120 groups of data collected from the literature [32], the factors that affect the frost resistance of recycled concrete mentioned in Table 1 are selected as input indexes, and the relative dynamic elastic modulus is taken as the output index to establish the sample dataset, which is divided into training set and test set. The training set contains 87.5% of data: that is, the training set contains 105 groups of data samples, while the test set contains 15 groups of test samples. The samples contained in the test set are marked in Appendix A. In order to obtain more accurate prediction results, it is necessary to optimize the initial input index of the network.

### 3.3. Sensitivity Analysis of Influencing Factors

As we all know, the accuracy of the prediction model is closely related to the dimensions of the input variables. If there are irrelevant or weakly correlated variables in the set of input variables, it is bound to increase the modeling time, reduce the accuracy of the model, and even result in over-fitting. Therefore, it is necessary to determine the importance of each input variable and eliminate irrelevant or weakly correlated input variables to improve the accuracy of model prediction. In this paper, grey correlation degree [33,34] is selected to evaluate the importance of each factor. The specific steps are as follows:

(1) Build the raw data matrix. In this paper, *i* group (*i* = 15) measured data of recycled concrete are used to analyze the parent factor (relative dynamic elastic modulus) and *j* (*j* = 9) sub-factor (mentioned in Table 1), then the original data matrix is obtained.

(1) $$\left[X\right]=[\begin{array}{l} x_{10}\\ x_{20}\\ \vdots \\ x_{i0} \end{array}\begin{array}{l} x_{11}\\ x_{21}\\ \vdots \\ x_{i1} \end{array}\begin{array}{l} \cdots \\ \cdots \\ \vdots \\ \cdots \end{array}{\begin{array}{l} x_{1j}\\ x_{2j}\\ \vdots \\ x_{ij} \end{array}]}_{15\times (9+1)}$$

(2) Unified dimension. In order to eliminate the impact of dimension, the homogenization method is used to carry out unified dimensional processing on the original data.

(2) $${\bar{x}}_{ij}=x_{ij}/\frac{i}{n}\sum_{i=1}^{n}x_{ij}$$

where, $x_{ij}$ is the value of a factor in the input layer, ${\bar{x}}_{ij}$ is the average value of the factor in the input layer, $x_{i0}$ is the relative dynamic elastic modulus value of the factor in the input layer after 200 times of freezing and thawing. *i =* 1, 2, *…*, *n*; *j =* 0, 1, 2, *…*, *m.*

(3) Calculate the absolute difference matrix and the maximum value.

(3) $$\left[\Delta \right]={\left|\delta_{ij}\right|}_{n\times (m+1)}$$

(4) $$\delta_{ij}=\left|{\bar{x}}_{ij}-{\bar{x}}_{i0}\right|$$

Then, the maximum value in the absolute difference matrix is:

(5) $$\Delta_{\min}=\min\left\{\delta_{ij}\right\}$$

(6) $$\Delta_{\max}=\max\left\{\delta_{ij}\right\}$$

(4) Solve the correlation coefficient matrix. The correlation coefficient matrix is calculated as follows:

(7) $$\left[L\right]={\left|l_{ij}\right|}_{n\times (m+1)}$$

(8) $$l_{ij}=\frac{\Delta_{\min}+\rho \Delta_{\max}}{\delta_{ij}+\rho \Delta_{\max}}$$

where, ρ is the resolution coefficient. The value of ρ is between 0 and 1. Generally, ρ = 0.5.

(5) Calculated correlation degree. In order to analyze the correlation between the parent factor and each sub-factor, it is necessary to calculate the correlation degree. The calculation formula is as follows:

(9) $$\gamma_{0i}=\frac{1}{n}\sum_{i=1}^{n}l_{ij}$$

where, $\gamma_{0i}$ is the correlation degree of the sub-factor $x_{i}$ to the parent factor $x_{0}$. A larger value of $\gamma_{0i}$ indicates a greater correlation.

### 3.4. BPNN Prediction and Accuracy Evaluation

**Figure 2:**
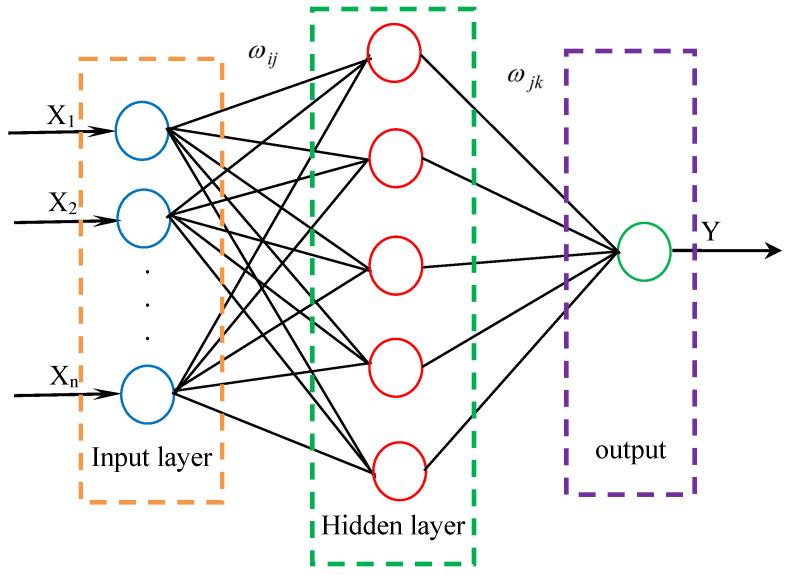
BPNN structure diagram.

A BPNN [27] is a typical multi-layer forward network, consisting of an input layer, an output layer and a hidden layer. The hidden layer can have one or more layers, and each layer is composed of multiple neurons. Its structure is shown in Figure 2. As can be seen from Figure 2, all connections are adopted between layers, and there is no mutual connection between the units of the same layer. Although neurons in the same layer cannot connect to each other, they can transmit data with neurons in another layer. The learning process of the BPNN algorithm consists of forward propagation and back propagation. In the forward propagation process, the input information from the input layer is processed through the hidden layer and then output in the output layer. Each layer of neurons only affects the neurons of next layer. If the expected output result is not obtained in the output layer, the error signal is returned along the original path, and the error is minimized by modifying the weights of neurons in each layer.

The following expressions can be used to express all processes that occur within the ANN framework [27]:

(10) $$Y=f_{sig}\{b_{0}+\sum_{j=1}^{n}[\omega_{j}\times f_{sig}(b_{nj}+\sum_{i=1}^{m}\omega_{ij}\delta_{i})]\}$$

where, *Y* is the output parameter, *b*_0_ is the offset term of the output layer, *n* is the number of neurons in the hidden layer, *j* represents a specific neuron in the hidden layer, $\omega_{j}$ is the connection weight between the *j*th hidden layer and a single output neuron, $\delta_{i}$ is the *i*th input variable and $f_{sig}$ is a nonlinear transfer function.

To effectively evaluate the accuracy of the two-stage prediction model proposed in this paper, determination coefficient (*R*^2^), mean square error (MSE), root mean square error (RMSE), mean absolute error (MAE) and mean absolute percentage error (MAPE) are used. Their expressions are shown as (11)–(15).

(11) $$R^{2}=1-\frac{\sum_{i=1}^{n}{\left(y_{i}-y_{i}^{\prime }\right)}^{2}}{\sum_{i=1}^{n}{\left(y_{i}-{\bar{y}}_{i}^{\prime }\right)}^{2}}$$

(12) $$MSE=\frac{\sum_{i=1}^{n}{\left(y_{i}^{\prime }-y_{i}\right)}^{2}}{n}$$

(13) $$RMSE=\sqrt{\frac{\sum_{i=1}^{n}{\left(y_{i}^{\prime }-y_{i}\right)}^{2}}{n}}$$

(14) $$MAE=\frac{1}{n}\sum_{i=1}^{n}\left|y_{i}^{\prime }-y_{i}\right|$$

(15) $$MAPE=\frac{1}{n}\sum_{i=1}^{n}\left|\frac{y_{i}^{\prime }-y_{i}}{y_{i}}\right|\times 100$$

where, $y_{i}$ is the *i*th test value, ${\bar{y}}_{i}$ is the average value of the *i*th test value, $y_{i}^{\prime }$ is the *i*th predicted value and ${\bar{y}}_{i}^{\prime }$ is the average of the *i*th predicted value.

## 4. Case Study

### 4.1. Sensitivity Analysis of Influencing Factors

**Figure 3:**
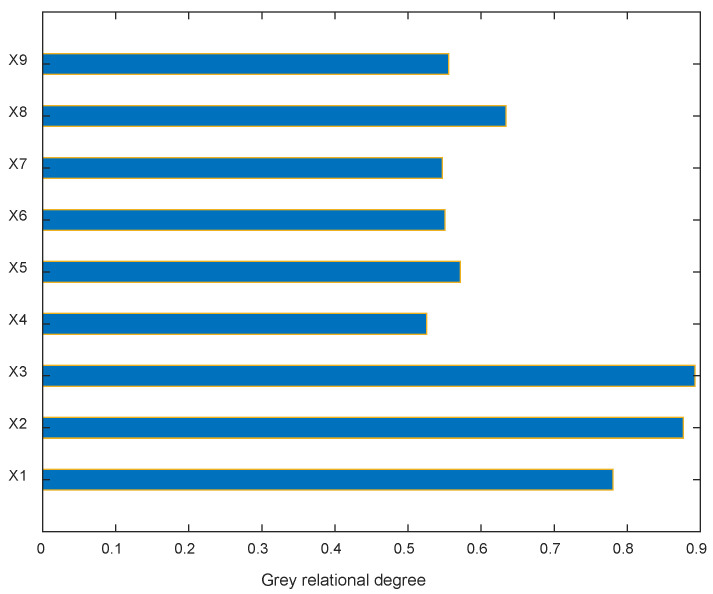
Sensitivity analysis results of influencing factors.

It can be seen from Table 1 in Section 2 that for the relative dynamic elastic modulus, the influencing factors are the quality of cement, water, sand, natural coarse aggregate, recycled aggregate, mineral powder, fly ash, air-entraining agent, fiber and the number of freeze–thaw cycles, so the initial frost-resistance-influencing indexes are 10. Since the number of freeze–thaw cycles is the influence factor of frost resistance that must be considered, which was mentioned in Section 2, sensitivity analysis is performed on the remaining nine factors. Taking the relative dynamic elastic modulus after 200 freeze–thawing sessions as an example, the grey correlation degree of each factor affecting the relative dynamic elastic modulus is calculated according to the steps described in Section 3.3, as shown in Table 2 and Figure 3.

It can be seen from Figure 3 that the correlation degree of cement, water, sand, recycled aggregate and air-entraining agent are all bigger than 0.57, and, meanwhile, natural coarse aggregate, mineral powder, fly ash and fiber are less than 0.57; it means these four ingredients have relatively little impact on the frost resistance of recycled concrete. Therefore, the original nine influencing factors of concrete ingredients are optimized into five, and considering the number of freeze–thaw cycles is the decisive factor for the freeze-resistance of concrete, six factors are used as input variables of the BPNN.

### 4.2. BP Network Structure Design

On the basis of grey correlation analysis, six input factors and one output factor (relative dynamic elastic modulus) including cement, water, sand, recycled aggregate, air-entraining agent and number of freeze–thaw cycles are determined. To eliminate the influence of different influencing factors on the learning accuracy and effect of a neural network, it is necessary to normalize the sample data before model training. The original data are normalized: that is, Formula (16) is used to convert the original value of the input layer to the value of the interval [0, 1], and finally the value is replaced by Formula (17) in the output layer.

(16) $${\bar{x}}_{i}=\frac{x_{i}-x_{\min}}{x_{\max}-x_{\min}}$$

(17) $$x_{i}={\bar{x}}_{i}(x_{\max}-x_{\min})+x_{\min}$$

where, ${\bar{x}}_{i}$ is the data after normalization processing, $x_{i}$ is the non-normalization data, $x_{\min}$ is the minimum value of the original data sample and $x_{\max}$ is the maximum value of the original data sample.

In this paper, a three-layer neural network with a single hidden layer is selected to predict the relative dynamic elastic modulus. There are six variables in the input layer, so the number of nodes in the input layer is six, and the output layer of the network has one variable, so the number of nodes is one. At present, there is no unified method to determine the number of nodes in the hidden layer of the network. In this paper, empirical formulas and sum of squares of error are used to confirm the number of nodes in the hidden layer. The common calculation formula for nodes in the hidden layer is shown in Equation (18) [35]

(18) $$S=\sqrt{n+m}+a$$

where *S* is the number of nodes in hidden layer, *n* is the number of input layer nodes, *m* is the number of output layer nodes, and a is an integer of [1,10], then the range of nodes in the hidden layer is:

(19) $$H=\sqrt{6+1}+[1,10]=[4,14]$$

**Figure 4:**
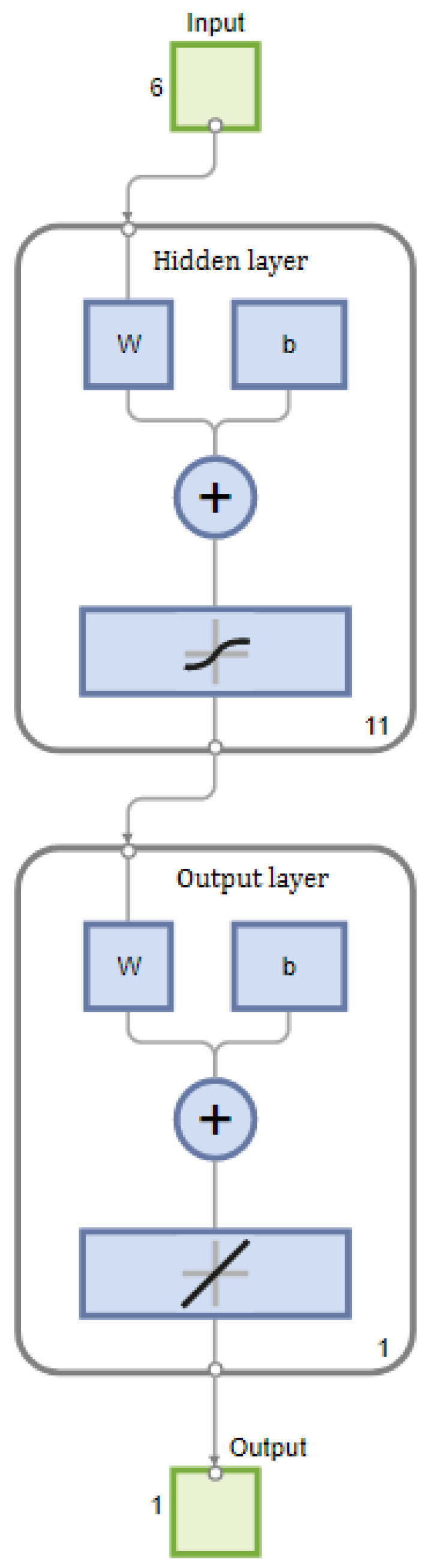
BP network diagram.

Therefore, the number of nodes in the hidden layer is finally determined to be 11 according to the minimum sum of squares error. So far, the structure of the BPNN used in this paper is 6-11-1, as shown in Figure 4. The transfer function of the hidden layer is tansig, and the transfer function of the output layer is purelin. The maximum of training steps is set to 1000, the training accuracy is set to 0.000001 and the learning rate is 0.01.

### 4.3. Results and Discussion

**Figure 5:**
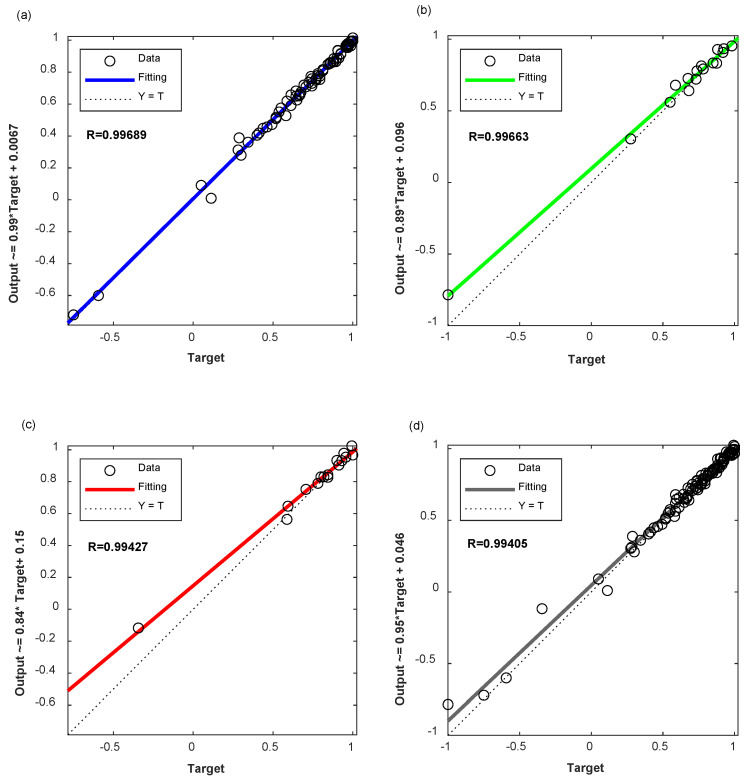
Establishment of two−stage BPNN model: (**a**) Training (**b**) Validation (**c**) Testing (**d**) all.

After the structure and parameters of the neural network are determined, the two-stage BPNN model proposed in this paper is used to predict the relative dynamic elastic modulus in the literature [32], the prediction results are compared with the experimental values are listed in Table 3. The input and output fitting curves of the two-stage BPNN model proposed in this paper in the stages of network training, verification and testing are shown in Figure 5.

It can be seen from Figure 5 that the correlation coefficient between the output values and the actual values of the BPNN is 0.99689 in the training process, 0.99663 in the verification process and 0.99427 in the testing process. For the establishment of the overall model, the correlation coefficient is 0.99405. It can also be seen from Figure 5 that the two-stage BPNN model proposed in this paper has high prediction accuracy, and the overall correlation coefficient is above 0.99.

**Figure 6:**
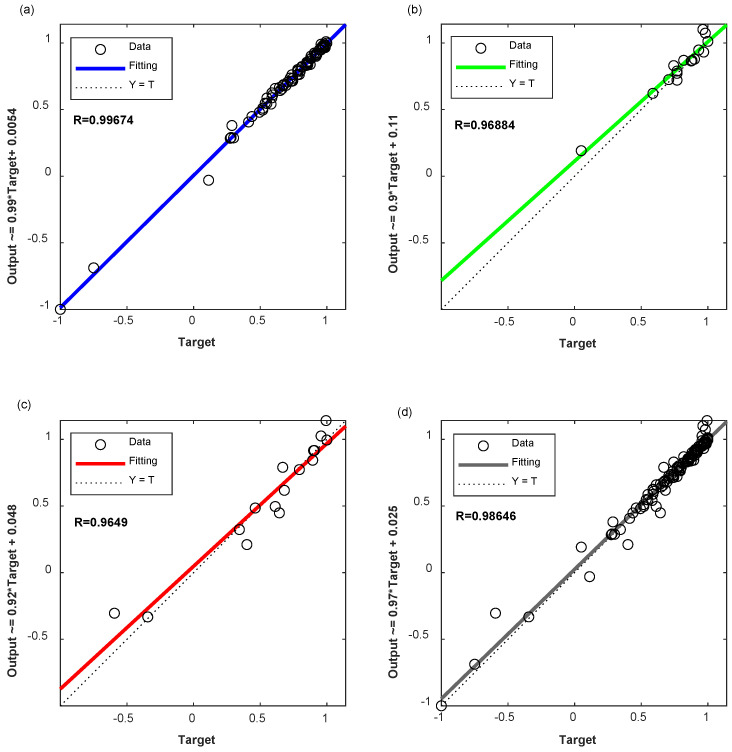
Establishment of BP neural network model: (**a**) Training (**b**) Validation (**c**) Testing (**d**) all.

**Figure 7:**
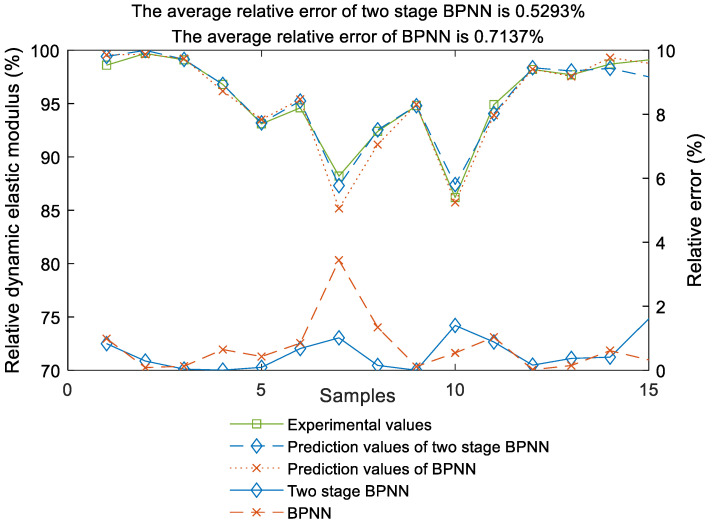
Comparison of prediction results.

In order to illustrate the superiority of the two-stage BPNN prediction model proposed in this paper, the comparison between two-stage BPNN and BPNN is also conducted in this paper. The input and output fitting curves of the training, verification and testing stages of the BPNN network are shown in Figure 6 and the prediction error comparison between these two models is shown in Figure 7. The establishment process of the BPNN model is exactly the same as that of the two-stage BPNN model, but its input vectors are 10 input vectors (9—mix proportion contents of concrete and 1—the number of freeze–thaw cycles) that are not optimized, the network structure is 10-12-1, and it also has a hidden layer. The number of hidden layer neurons is determined by the same method of two-stage BPNN.

It can be seen from Figure 6 that the correlation coefficient between the output values and the actual values of the BPNN is 0.99674 in the training process, 0.96884 in the verification process, and 0.96490 in the test process. For the establishment of the overall model, the correlation coefficient is 0.98646. By comparing Figure 5 and Figure 6, it can be seen that the two-stage BPNN model proposed in this paper has higher prediction accuracy and higher correlation than the BPNN in training, verification and testing stages.

As can be seen from Figure 7, the average relative error of the two-stage BPNN prediction model proposed in this paper is 0.5293%, while the average relative error of the BPNN prediction model is 0.7137%. Obviously, the prediction accuracy of the two-stage BPNN prediction model proposed in this paper is higher than that of the BPNN prediction model. Moreover, the error fluctuation of the two-stage BPNN prediction model is very stable, and it fluctuates in a small range, and the prediction accuracy is high.

To further verify the performance of the two-stage BPNN model proposed in this paper, MAE, MSE, RMSE, MAPE, R^2^ and run time are used respectively to evaluate these two network models. The results are shown in Table 4.

It can be seen from Table 4 that all evaluation indexes of the two-stage BPNN prediction model proposed in this paper, including MAE, MSE, RMSE, MAPE, R^2^ and network run time, are better than the BPNN: it is mainly because after the grey sensitivity analysis of influencing factors, the input variables of the network are simplified, thus shortening the run time of the neural network. The fitting degree of two-stage BPNN model to the data is improved, so the prediction accuracy is higher.

## 5. Conclusions

(1) This paper proposes a two-stage BPNN model. Benefitting from the database collected from the literature [32], two prediction models based on a two-stage BPNN model and a BPNN for relative dynamic elastic modulus prediction of recycled-aggregate concrete with active mixture were established. Compared with BPNN, the proposed two-stage BPNN model has better performance, better prediction accuracy and shorter run time.
(2) The frost resistance of recycled concrete with active admixture is affected by many factors under freeze–thaw cycles. Using the two-stage frost resistance prediction model proposed in this paper, with cement, water, sand, recycled aggregate, air-entraining agent and the number of free–thaw cycles as input variables and relative dynamic elastic modulus as output variables, the dilemma of establishing an accurate mathematical theoretical model is avoided, benefiting from the powerful nonlinear mapping ability of the neural network. Through sensitivity analysis, the input vector of the neural network is reduced, thus improving the prediction accuracy and run time of the neural network.
(3) In the further research, we will collect more test data to expand our database, and plan to combine it with other network models such as deep learning networks to predict the frost resistance of concrete, so as to improve the generalization ability of the prediction model.

## Figures and Tables

**Table 1:** The change range of original input and output data in [32].

	Mix Proportion									Freeze-Thaw Cycles	Relative Dynamic Elastic Modulus (200th Freeze-Thaw)
	Cement (X_1_)	Water (X_2_)	Sand (X_3_)	Natural Coarse Aggregate (X_4_)	Recycled Coarse Aggregate (X_5_)	Mineral Powder (X_6_)	Fly Ash (X_7_)	Air Entraining Agent (X_8_)	Fiber (X_9_)		
Minimum	128	153	655	0	0	0	0	0	0	25	67.8
Maximum	321	183	683	1146	1146	96.3	125.2	0.032	1.5	200	99.8

**Table 2:** Correlation degree of influencing factors.

	Cement (X_1_)	Water (X_2_)	Sand (X_3_)	Natural Coarse Aggregate (X_4_)	Recycled Coarse Aggregate (X_5_)	Mineral Powder (X_6_)	Fly Ash (X_7_)	Air Entraining Agent (X_8_)	Fiber (X_9_)
Grey correlation degree	0.7804	0.8766	0.8928	0.5254	0.5717	0.5507	0.5469	0.6340	0.5558

**Table 3:** The predicted and experimental values of relative dynamic elastic modulus.

Input										Output		
X_1_	X_2_	X_3_	X_4_	X_5_	X_6_	X_7_	X_8_	X_9_	Freeze-Thaw Cycles	Relative Dynamic Elastic Modulus (%)		
										Experimental Values [32]	Prediction by Two Stage BPNN	Prediction by BPNN
289	165	675	688	458	0	41.7	0.016	0.5	50	98.6	99.42	99.57
257	174	664	344	802	0	83.5	0.024	1.0	25	99.7	99.99	99.62
225	183	655	0	1146	0	125.2	0.032	1.5	75	99.1	99.14	99.22
289	183	683	0	1146	32.1	0	0.016	1.0	100	96.8	96.80	96.17
257	174	675	344	802	32.1	41.7	0	1.5	125	93.1	93.19	93.50
225	165	664	688	458	32.1	83.5	0.032	0	175	94.6	95.24	95.40
193	153	655	1146	0	32.1	125.2	0.024	0.5	200	88.2	87.31	85.17
257	165	683	688	458	64.2	0	0.024	1.5	200	92.4	92.54	91.16
225	153	675	1146	0	64.2	41.7	0.032	1.0	175	94.8	94.80	94.91
193	183	664	0	1146	64.2	83.5	0	0.5	150	86.2	87.41	85.73
161	174	655	344	802	64.2	125.2	0.016	0	125	94.9	94.06	93.92
321	174	683	344	802	96.3	0	0.032	0.5	100	98.2	98.36	98.22
193	183	675	0	1146	96.3	41.7	0.024	0	75	97.7	98.06	97.55
161	153	664	1146	0	96.3	83.5	0.016	1.5	50	98.7	98.30	99.31
128	165	655	688	458	96.3	125.2	0	1	25	99.1	97.51	98.78

Note: X_1_, X_2_, X_3_, X_5_ and X_8_ are the input vectors of proposed two stage BPNN.

**Table 4:** Performance comparison between the two models.

Model	MAE	MSE	RMSE	MAPE (%)	R^2^	Run Time (s)
Two-stage BPNN	0.49891	0.46836	0.68437	0.52938	0.96998	41.803
BPNN	0.66249	0.97266	0.98624	0.71365	0.93792	72.372

## Data Availability

All the data are available in the tables and can be traced back to references cited in this paper.

## Appendix A

**Table A1:** The Data for Training and Testing the BPNN. Marked with * at the Serial Numbers Are Those for Testing.

No.	X_1_	X_2_	X_3_	X_4_	X_5_	X_6_	X_7_	X_8_	X_9_	Freeze-Thaw Cycles	Relative Dynamic Elastic Modulus
1	289	165	675	688	458	0	41.7	0.016	0.5	25	99.5
2 *	289	165	675	688	458	0	41.7	0.016	0.5	50	98.6 *
3	289	165	675	688	458	0	41.7	0.016	0.5	75	97.9
4	289	165	675	688	458	0	41.7	0.016	0.5	100	97.3
5	289	165	675	688	458	0	41.7	0.016	0.5	125	96.5
6	289	165	675	688	458	0	41.7	0.016	0.5	150	94.1
7	289	165	675	688	458	0	41.7	0.016	0.5	175	93.6
8	289	165	675	688	458	0	41.7	0.016	0.5	200	90.2
9 *	257	174	664	344	802	0	83.5	0.024	1.0	25	99.7 *
10	257	174	664	344	802	0	83.5	0.024	1.0	50	98.9
11	257	174	664	344	802	0	83.5	0.024	1.0	75	98.3
12	257	174	664	344	802	0	83.5	0.024	1.0	100	97.9
13	257	174	664	344	802	0	83.5	0.024	1.0	125	97.3
14	257	174	664	344	802	0	83.5	0.024	1.0	150	96.1
15	257	174	664	344	802	0	83.5	0.024	1.0	175	94.6
16	257	174	664	344	802	0	83.5	0.024	1.0	200	92.6
17	225	183	655	0	1146	0	125.2	0.032	1.5	25	99.8
18	225	183	655	0	1146	0	125.2	0.032	1.5	50	99.4
19 *	225	183	655	0	1146	0	125.2	0.032	1.5	75	99.1 *
20	225	183	655	0	1146	0	125.2	0.032	1.5	100	98.7
21	225	183	655	0	1146	0	125.2	0.032	1.5	125	98.4
22	225	183	655	0	1146	0	125.2	0.032	1.5	150	96.9
23	225	183	655	0	1146	0	125.2	0.032	1.5	175	95.7
24	225	183	655	0	1146	0	125.2	0.032	1.5	200	94.5
25	289	183	683	0	1146	32.1	0	0.016	1.0	25	99.5
26	289	183	683	0	1146	32.1	0	0.016	1.0	50	98.4
27	289	183	683	0	1146	32.1	0	0.016	1.0	75	97.5
28 *	289	183	683	0	1146	32.1	0	0.016	1.0	100	96.8 *
29	289	183	683	0	1146	32.1	0	0.016	1.0	125	96.1
30	289	183	683	0	1146	32.1	0	0.016	1.0	150	93.6
31	289	183	683	0	1146	32.1	0	0.016	1.0	175	93.1
32	289	183	683	0	1146	32.1	0	0.016	1.0	200	88.3
33	257	174	675	344	802	32.1	41.7	0	1.5	25	99.3
34	257	174	675	344	802	32.1	41.7	0	1.5	50	98.5
35	257	174	675	344	802	32.1	41.7	0	1.5	75	96.8
36	257	174	675	344	802	32.1	41.7	0	1.5	100	95.7
37 *	257	174	675	344	802	32.1	41.7	0	1.5	125	93.1 *
38	257	174	675	344	802	32.1	41.7	0	1.5	150	88.4
39	257	174	675	344	802	32.1	41.7	0	1.5	175	85.6
40	257	174	675	344	802	32.1	41.7	0	1.5	200	71.8
41	225	165	664	688	458	32.1	83.5	0.032	0	25	99.8
42	225	165	664	688	458	32.1	83.5	0.032	0	50	99.3
43	225	165	664	688	458	32.1	83.5	0.032	0	75	99.1
44	225	165	664	688	458	32.1	83.5	0.032	0	100	98.6
45	225	165	664	688	458	32.1	83.5	0.032	0	125	98.2
46	225	165	664	688	458	32.1	83.5	0.032	0	150	96.8
47 *	225	165	664	688	458	32.1	83.5	0.032	0	175	94.6 *
48	225	165	664	688	458	32.1	83.5	0.032	0	200	93.2
49	193	153	655	1146	0	32.1	125.2	0.024	0.5	25	99.6
50	193	153	655	1146	0	32.1	125.2	0.024	0.5	50	98.2
51	193	153	655	1146	0	32.1	125.2	0.024	0.5	75	98.1
52	193	153	655	1146	0	32.1	125.2	0.024	0.5	100	95.6
53	193	153	655	1146	0	32.1	125.2	0.024	0.5	125	94.4
54	193	153	655	1146	0	32.1	125.2	0.024	0.5	150	92.1
55	193	153	655	1146	0	32.1	125.2	0.024	0.5	175	89.3
56 *	193	153	655	1146	0	32.1	125.2	0.024	0.5	200	88.2 *
57	257	165	683	688	458	64.2	0	0.024	1.5	25	99.7
58	257	165	683	688	458	64.2	0	0.024	1.5	50	99.1
59	257	165	683	688	458	64.2	0	0.024	1.5	75	98.6
60	257	165	683	688	458	64.2	0	0.024	1.5	100	97.3
61	257	165	683	688	458	64.2	0	0.024	1.5	125	96.5
62	257	165	683	688	458	64.2	0	0.024	1.5	150	95.1
63	257	165	683	688	458	64.2	0	0.024	1.5	175	94.7
64 *	257	165	683	688	458	64.2	0	0.024	1.5	200	92.4 *
65	225	153	675	1146	0	64.2	41.7	0.032	1.0	25	99.8
66	225	153	675	1146	0	64.2	41.7	0.032	1.0	50	99.2
67	225	153	675	1146	0	64.2	41.7	0.032	1.0	75	98.6
68	225	153	675	1146	0	64.2	41.7	0.032	1.0	100	97.5
69	225	153	675	1146	0	64.2	41.7	0.032	1.0	125	96.3
70	225	153	675	1146	0	64.2	41.7	0.032	1.0	150	95.4
71 *	225	153	675	1146	0	64.2	41.7	0.032	1.0	175	94.8 *
72	225	153	675	1146	0	64.2	41.7	0.032	1.0	200	94.2
73	193	183	664	0	1146	64.2	83.5	0	0.5	25	99.1
74	193	183	664	0	1146	64.2	83.5	0	0.5	50	97.8
75	193	183	664	0	1146	64.2	83.5	0	0.5	75	95.7
76	193	183	664	0	1146	64.2	83.5	0	0.5	100	92.6
77	193	183	664	0	1146	64.2	83.5	0	0.5	125	90.4
78 *	193	183	664	0	1146	64.2	83.5	0	0.5	150	86.2 *
79	193	183	664	0	1146	64.2	83.5	0	0.5	175	78.3
80	193	183	664	0	1146	64.2	83.5	0	0.5	200	67.8
81	161	174	655	344	802	64.2	125.2	0.016	0	25	99.2
82	161	174	655	344	802	64.2	125.2	0.016	0	50	97.9
83	161	174	655	344	802	64.2	125.2	0.016	0	75	96.3
84	161	174	655	344	802	64.2	125.2	0.016	0	100	95.5
85 *	161	174	655	344	802	64.2	125.2	0.016	0	125	94.9 *
86	161	174	655	344	802	64.2	125.2	0.016	0	150	92.4
87	161	174	655	344	802	64.2	125.2	0.016	0	175	90.8
88	161	174	655	344	802	64.2	125.2	0.016	0	200	88.6
89	321	174	683	344	802	96.3	0	0.032	0.5	25	99.7
90	321	174	683	344	802	96.3	0	0.032	0.5	50	99.6
91	321	174	683	344	802	96.3	0	0.032	0.5	75	99.3
92 *	321	174	683	344	802	96.3	0	0.032	0.5	100	98.2 *
93	321	174	683	344	802	96.3	0	0.032	0.5	125	97.6
94	321	174	683	344	802	96.3	0	0.032	0.5	150	96.3
95	321	174	683	344	802	96.3	0	0.032	0.5	175	94.9
96	321	174	683	344	802	96.3	0	0.032	0.5	200	94.1
97	193	183	675	0	1146	96.3	41.7	0.024	0	25	99.6
98	193	183	675	0	1146	96.3	41.7	0.024	0	50	98.3
99 *	193	183	675	0	1146	96.3	41.7	0.024	0	75	97.7 *
100	193	183	675	0	1146	96.3	41.7	0.024	0	100	96.6
101	193	183	675	0	1146	96.3	41.7	0.024	0	125	95.1
102	193	183	675	0	1146	96.3	41.7	0.024	0	150	94.5
103	193	183	675	0	1146	96.3	41.7	0.024	0	175	93.2
104	193	183	675	0	1146	96.3	41.7	0.024	0	200	91.2
105	161	153	664	1146	0	96.3	83.5	0.016	1.5	25	99.2
106 *	161	153	664	1146	0	96.3	83.5	0.016	1.5	50	98.7 *
107	161	153	664	1146	0	96.3	83.5	0.016	1.5	75	97.4
108	161	153	664	1146	0	96.3	83.5	0.016	1.5	100	96.3
109	161	153	664	1146	0	96.3	83.5	0.016	1.5	125	94.6
110	161	153	664	1146	0	96.3	83.5	0.016	1.5	150	93.3
111	161	153	664	1146	0	96.3	83.5	0.016	1.5	175	92.1
112	161	153	664	1146	0	96.3	83.5	0.016	1.5	200	88.2
113 *	128	165	655	688	458	96.3	125.2	0	1	25	99.1 *
114	128	165	655	688	458	96.3	125.2	0	1	50	97.8
115	128	165	655	688	458	96.3	125.2	0	1	75	96.1
116	128	165	655	688	458	96.3	125.2	0	1	100	95.6
117	128	165	655	688	458	96.3	125.2	0	1	125	93.2
118	128	165	655	688	458	96.3	125.2	0	1	150	91.7
119	128	165	655	688	458	96.3	125.2	0	1	175	84.6
120	128	165	655	688	458	96.3	125.2	0	1	200	74.3
